# The Views of Healthcare Professionals on iFall, a Smartphone Application for Falls Reporting in Parkinson’s Disease: A Qualitative Study

**DOI:** 10.1177/08919887251317728

**Published:** 2025-02-01

**Authors:** Michael C. Kelly, Jenni Naisby, Jill Wales, Elaine Webster, Gerry Standerline, Gill Barry, Annee Amjad, Jason Moore, Natasha Ratcliffe, Alan Godfrey, Rosie Morris

**Affiliations:** 1 Department of Sport, Exercise and Rehabilitation, 5995 Northumbria University, Newcastle-upon-Tyne, UK; 2 Person with Parkinson’s, Co-Researcher, UK; 3 9097 Parkinson’s UK, London, UK; 4 Department of Computer and Information Sciences, 5995 Northumbria University, Newcastle-upon-Tyne, UK

**Keywords:** Parkinson’s disease, co-design, falls, digital measurement tool

## Abstract

**Background:** Accurate falls reporting is important in the management of Parkinson’s disease. One way in which to improve accuracy is by providing a smartphone app to log fall events. This qualitative study sought to gain insights from healthcare professionals based in the United Kingdom on a novel smartphone application co-developed by people with Parkinson’s (PwP) disease for falls reporting. **Research Methods:** A purposive sample of n=11 healthcare professionals with expertise in Parkinson’s were recruited to take part in a focus group to explore their views on the smartphone app. Framework analysis was utilised to interpret the data. **Results:** Participants discussed the applications role in clinical practice, research, and provided recommendations for future improvements. Within the overarching theme of implementation of iFall in clinical and research practice, three subthemes emerged: (1) applicability to clinical practice, (2) the future of iFall in research and (3) future developments. The application was viewed positively, exhibiting potential to address important contemporary issues within falls reporting and research, whilst being clear, simple and easy to use. Implementation challenges of the application, such as IT integration were highlighted, while enhancements such as voice recognition were suggested. **Conclusions:** Incorporating suggestions from healthcare professionals and piloting of the application with PwP will increase the likelihood of successful implementation of the iFall app into clinical practice and research.

## Plain Language Summary

For people with Parkinson’s, there is an increased chance of falling. Falling can cause injury, anxiety, pain and a worsening of quality of life. Being able to track falls is important for healthcare professionals to be able to help support people with Parkinson’s. Measuring falls can be difficult. Often paper diaries are used, but this can be difficult and time consuming for people with Parkinson’s to complete. A smartphone application has been developed by people with Parkinson’s to help record falls. We wanted to understand what healthcare professionals who work with people with Parkinson’s thought of the application and how it could help inform clinical practice and research. We also wanted to know how it could be improved further. We used three focus groups, with eleven healthcare professionals, and they provided their thoughts on the application. The healthcare professionals felt that the application was very clear and simple to use and had potential to help clinician’s and researchers improve how falls are recorded. They highlighted areas that could be improved, such as adding more options to add more details about the falls. They also highlighted some obstacles to putting the application into practice, which we will use to improve how we can use the application in the future.

## Background

Falls in people with Parkinson’s disease (PwP) are common. PwP are 2-4 times more likely to fall compared to age-matched healthy older adults.^1^ Falls in PwP result in increased anxiety, reduced independence, and reduced quality of life.^2^ Furthermore, falls are one of the main causes of hospital admissions within this population, leading to high healthcare costs.^3^ Treatments for falls in PwP are currently limited and have a minimal response to dopaminergic medication, with Physiotherapy and exercise demonstrating benefits for reducing falls.^4-7^

The causes of falls in PwP are multifactorial, with over 30 risk factors for falls previously identified.^8^ Understanding the cause of falls in PwP is critical as this likely determines the route of treatment or type of rehabilitation provided.^8^ Determining the cause of falls in patients can take a significant amount of clinical time and relies on subjective reporting. Due to the high number of falls and cognitive impairment in PwP, patient recall likely leads to underestimation of falls incidence in this population.^9^ In clinical practice, there is no established or standardised method for recording falls that occur in daily life, however patients may be asked to keep a log of fall events. In research projects, the current gold standard methodology is pen and paper diaries, however paper-based diaries have high-cost implications both in money and resource allocation.^10^ Paper based diaries also have issues relating to handwriting.^11^ Therefore, a more standardised way of reporting falls incidence with improved ecological validity is required both for clinical practice and research.

We previously developed a novel smartphone application for reporting falls and near-misses in PwP.^12^ The app was co-designed by researchers, healthcare professionals and people affected by Parkinson’s. Once developed, the app underwent beta-testing in 15 PwP to determine the usability and acceptability. The app was determined to be easy to use, of interest to PwP, and of good quality. PwP reported that the app would be useful as a self-management tool and that they would use the app alongside their healthcare professional to further inform their Parkinson’s care.^12^ However, this study did not determine how healthcare professionals would use the app in their clinical practice, or whether it would be a useful digital measurement tool for implementing into clinical research trials. To implement the app appropriately, we need to further understand how the app would be best utilised by healthcare professionals and researchers to improve their practice.

The aim of this project was to gain insights from healthcare professionals with expertise in PD on the current prototype of the iFall smartphone application. We aimed to investigate 1) the role of the smartphone app in future clinical practice, 2) the role of the smartphone application in research practice for reporting falls, and 3) modifications that could be made to future iterations of the smartphone application based on their clinical expertise.

## Method

### Study Design

A qualitative study design, utilising three focus groups (FG). The study aimed to explore the views of clinicians and researchers, in relation to a digital falls reporting smart phone application, the iFall app, which was coproduced with PwP.^12^ In brief, the app was designed by both researchers and people affected by Parkinson’s (PwP, family members, carers) to allow PwP to log a fall or a near-miss of a fall in a smartphone app, instead of a paper-based log. The app was designed so that it was easy to log a fall, the cause and location of a fall, the user could also download a report of their falls and near-misses, see Supplemental File 1 for an image of App. The details of the co-design and usability have been published previously.^12^

**Figure 1.**
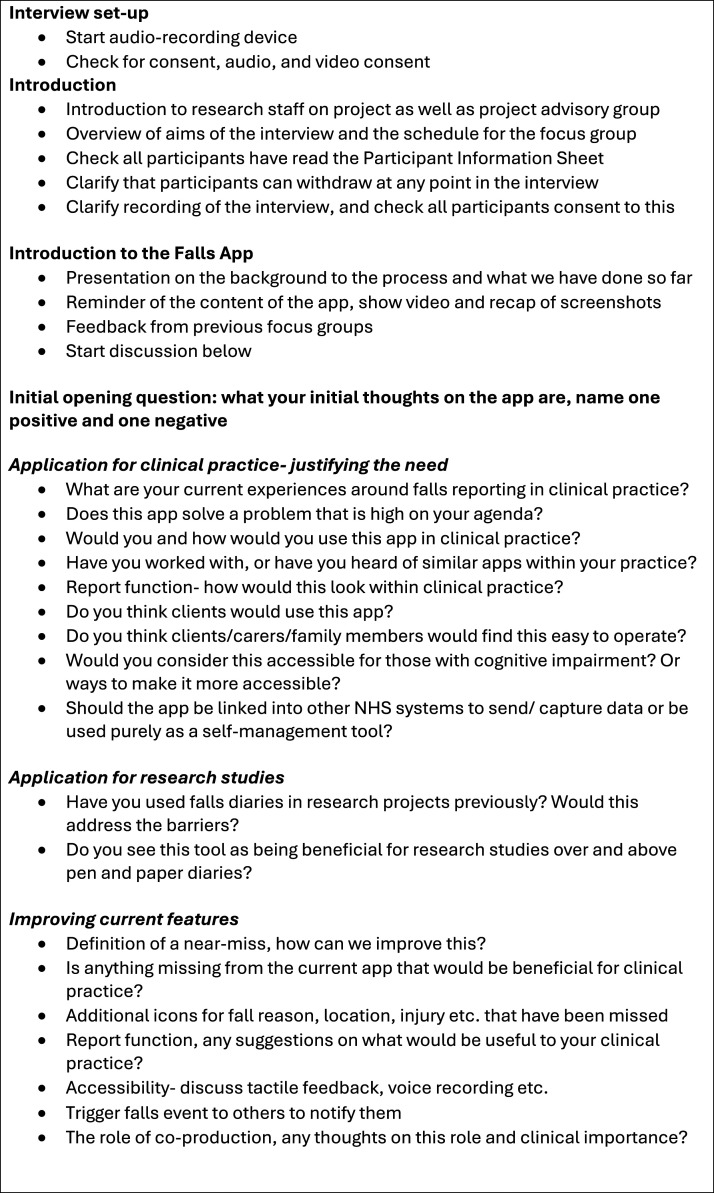
Interview guide.

An interview guide (Figure 1) was developed by authors RM, EW and GS with questions that aimed to understand the participants views on how the app could be utilised in clinical practice, future research and how the app could be improved. RM is an academic physiotherapist and EW and GS are people with lived experience of Parkinson’s. Findings from interviews with PwP^12^ also informed the interview guide. Prior to the FG, each participant had been given a presentation relating to the iFall app, which included details on the purpose of the app, the current design and feedback from PwP,^12^ so that they could familiarise themselves with the content, and utilisation of the app. This study is reported using the Standards for Reporting Qualitative Research (SRQR).^13^

### Participants

A purposive sample including 11 clinicians and clinical academics were recruited to the study consisting of three focus groups (two, four and five participants per group respectively). Participants were required to be a registered healthcare professional, with clinical and/or research experience in PwP. Participants were recruited via Parkinson’s UK and current networks of the researcher (RM). Participants were provided with a participant information sheet including details of the study during recruitment, which included the aims of the study. Participants provided consent via email and consented to the online recording of the FG.

Participants had a range of clinical and research roles, as well as experience of working with PwP, and were located across the United Kingdom (Table 1). Participants were allocated to each FG based on availability. In total, the focus groups lasted 220 minutes, averaging 73 min per focus group, ranging from 57 to 85 min.

**Table 1.** Participant Details.

Participant	Role	Experience with PD (years)	Focus group
1	Nurse	25 (clinal and research)	3
2	Physiotherapist	5.5	3
3	Physiotherapist	6 (5 yrs clinically, 1 yr. PD specific research)	2
4	Physiotherapist	5 yrs research + 10 yrs clinical neuro-rehab inc PD (NHS & Private)	2
5	Doctor	27	2
6	Physiotherapist	>10	2
7	PD Nurse Specialist	4 (1 year Nurse Specialist 3 years District Nursing Sister)	1
8	Parkinson’s Nurse Specialist	8	1
9	Registered Mental Health Nurse	6	1
10	Physiotherapist	18	1
11	Physiotherapist	25	1

### Statement of Ethical Approval

Ethical approval was granted by Northumbria University Ethical committee (Ref. 35604), in March 2022, prior to commencement of the study.

### Data Collection

The FG took place between May and June 2022, using teleconferencing and was conducted by RM, EW and GS. Each FG was recorded using Microsoft teams (MS Teams). At the start of each interview, RM provided an overview of the iFall app to help provide an aide memoir for the participants and to help facilitate depth to each FG discussion. Each FG was recorded via MS Teams.

### Analysis

Framework analysis^14^ was utilised, and conducted by MK and JN. Framework analysis consists of seven stages, and is a form of thematic analysis^14^ and therefore, not tied to a specific discipline or construct.^15^ Framework analysis can be utilised as part of an evaluative process,^16,17^ therefore was suitable to support the aims of this study.

All of the FG were independently transcribed verbatim using a professional transcriber. The first FG was coded by MK and JN independently to develop an analytical framework. Both MK and JN met to compare and discuss the codes, and following this, using Nvivo 12 (Lumivero, QSR international) an analytical framework was developed, then applied to each of the subsequent transcripts. The data was charted into a framework matrix and finally interpreted by MK and JN. During the analysis, MK and JN frequently discussed the development and application of the framework and allowed new codes to be developed as the analysis progressed.

## Results

**Figure 2.**
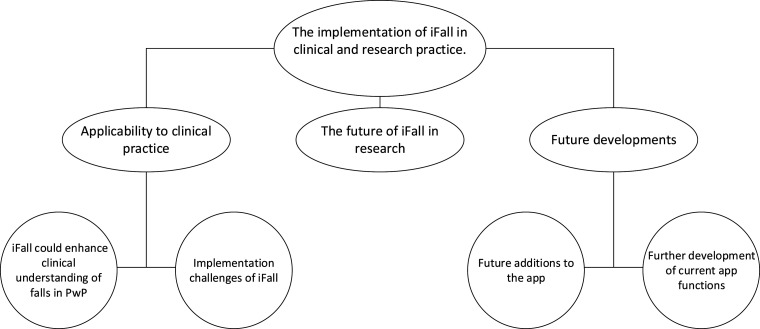
Themes and subthemes of the study.

Following the framework analysis, the implementation of iFall in clinical and research practice was the prevailing overarching theme (Figure 2). Three subthemes emerged about the iFall app, namely, 1) Applicability to clinical practice 2) The future of iFall in research 3) Future developments. Two further subthemes emerged within applicability to clinical practice and future developments.

### Applicability to Clinical Practice

#### iFall Could Enhance Clinical Understanding of Falls in PwP

Within current practice, drawbacks to measurement and recording falls were identified. These drawbacks were predominantly relating to how (in)frequently patients would complete paper-based diaries, but also with the accuracy of falls recording. It was highlighted that falls diaries were infrequently completed, and they found that the recollection of the details of the falls was at times lacking. For example, changes in gait, were rarely discussed by patients in detail. Due to the nature of PD, communication can be difficult for patients, this therefore provides a challenge for clinicians to gain detailed information relating to a fall, and impacts on what they would be able to cover within an appointment.

*It can be quite a tricky area because often if there's been a significant fall… There's a lot of story around it, and so you can have a 20-minute appointment with a… With a patient, and 15 minutes can be spent with them describing the fall. And when you're trying to get to other things, that's quite time consuming. So, you need to hear about it. But there was certainly… When I was… When I had that experience of people coming in, either in community setting or in outpatients, it could take the entire session, just hearing the story around that fall* (R4).

When discussing the drawbacks of current practice, the participants referred to the roles in which the iFall app could play within clinical practice. The app was viewed positively, including the overall concept whereby the app could be used to record falls, but also how the app could support clinical practice (as well as research) in its current version.

The ability to gain details about the falls was viewed very favourably by the participants. Being able to gain insight into how a patient falls, the environment or circumstances involved around the fall, as well as the outcome of the fall, from both a physical and emotional perspective were deemed as beneficial. Gaining this level of detail would be able to support the management of the patient, from multiple professions, providing holistic management. Understanding the outcome or consequence of a fall, was also deemed important by the participants, alongside the severity of the fall.

*You know, that’s the frustrating bit. And, again, you can’t then work out is it part of wearing off, it is part of...? You know, is it a functional thing? Is it an OH thing? You know, even asking about loss of consciousness or blacking out - often people can’t remember even that kind of thing. Or feeling dizzy or lightheaded* (R1).

The emotional aspect, including fear and anxiety was also discussed and was deliberated as something that could be linked to an increased risk for falls in the future. Understanding these potential risk factors could help the professional work with the person with Parkinson’s.

*I would say confusion would be a really good one to bring in, or feeling confused. I don't know why it happened. Because that, for me, is the flag because that's what adds the anxiety. Because if someone can explain away, oh I tripped over the carpet or I tripped over the cat or the teenager’s… Whatever. But when they don't know why it's happened… You know, I don't understand why it happened. That's what triggers that… For the people that I work with, that anxiety. Because they don't know how to predict it again* (R4).

The environment within which the person with Parkinson’s had fallen was a key factor. This information could be used to help explain why falls may be occurring and could be used to help patients reduce the risk of falling.

* From an MDT point of view, one of the things that my occupational therapy colleagues used to really struggle with is if someone would come into clinic, and I’d be talking to them at falls and stuff, and then we'd get them… Ask them to go and do an environmental assessment. I think having that extra bit of information of this is where the falls happen would really, sort of, help those discussions about actually we really do need to make some adaptations around here* (R3).

Accurate, detailed and condition specific information, such as freezing was a key positive of the iFall app. Understanding the details of a fall, such as where they were falling, the direction they fell, was considered vital to guiding a plan to help them as individuals from the outset:

*Because when I’m doing my assessments with new… You know, especially with new people that come into me - really getting down to the, okay, when you’re falling, how is it? How are you falling? Because that’s going to shape my rehab and help me think about what exercises we’re going to do and… … I think that is just so, so vital and what this app is going to be awesome for *(R3)*.*

While discussing the benefits that the app could bring to clinical practice, the participants highlighted how that the data from the app could be utilised to aid communication as well as provide an opportunity to educate the patient about their falls. This could be used in turn to help patients recognise factors that previously led to a fall, and guide the patient making changes that could potentially reduce the risk of future falls:

*And I really like your... You know, your report - because then you could really see that. And then that would really support the person with Parkinson’s to see, oh yeah, I’m falling when I do this particularly. Or when I’m going there. Or in this location *(R1)*.*

The app was viewed positively in terms of its user-friendly nature, which was visually accessible and pleasing. The presentation of the app was considered appropriate for both patients and clinicians, while being considered as self-explanatory:

*it looks amazing and really professional, and really quite clear - the screens aren’t too cluttered… *(R6)

An overriding potential benefit of the app, which was discussed throughout all focus groups, was the desire to have the information collected by the app to be provided before an appointment. This could be through a variety of means, such as a report, or print out. Although this was not built into the app, the notion of having the information prior to an appointment was deemed to be highly desirable by the clinicians.

*But getting people to fill out, kind of, outcome measures questionnaires before they even come to clinic - send it via MS Forms and stuff - getting them to send it in. If we could be sending, you know, the quality of life and wellbeing questionnaires, and then asking for their report at the same time, it would just be… Be fantastic. You know, you could be coming into clinic and actually be prepared rather than, sort of, spending ages, you know, going through it and stuff like that* (R3).

Ultimately, the participants felt that the app as an overall concept could be a valuable addition to clinical practice, fulfilling a need where there is a gap in current practice, and a step towards supporting better care for patients.

*But positives, I think, definitely this is a space that we need to move into. Because you can see how important it is for people to understand their symptoms and to be able to get help when they need it. So, I think that’s really important that we give them a tool like this. So, I think it’s a lot of positives more than anything* (R2).

#### Implementation Challenges of iFall

A key discussion point related to the terminology utilised when discussing near misses. Each focus group discussed the importance of a definition of a near miss. Not only was having a clear definition important, but the participants felt that information about a near miss would be beneficial to know. For example, understanding if patients could save themselves from a fall, using the wall or furniture, could be used to help pre-empt falls, as opposed to waiting for them to happen. Participants felt recording a near miss was important, but recognised an issue with a lack of definition:

*But I think one thing that we're probably not so great on is following up with the, okay, has there been any near misses…You know, there hadn't really been a consensus over what are near miss is…*

*…So I'd be really interested to know what's… You know, what was…? What was the kind of the…? Was there any consensus at all? What were people considering as near miss and how are we…? How are we going to address that? Because I think, actually, from my point of view, if someone is falling, yes, it's important to know… You know, the patterns and stuff. But actually the near misses and why they're starting to happen and… And really getting in there, and that to then encourage people to start to do preventative exercise and preventative therapy, rather than reactive, would be fantastic* (R3).

Whilst having an ability to collect a range of data, they recognised the potential of overloading the app for users. There was a balance between having enough information, but not losing sight of what the main benefits of the app could be- namely recording falls.

*But, you know, it’s just like a screener for me. So, some of that would need more detailed assessment afterwards. But you wouldn’t want to do all that with the app. Because, as you say, it would be overburdensome* (R1).

Participants were also cognisant of the importance that the app was able, as well as available, to support a wide range of patients. Accessibility and inclusivity of the app, for all patients, regardless of their socio-economic status or stage of PD was important for the participants, and they discussed where there could be issues for users in these areas. For example, not all PwP may have access to a smart phone to install the app, or have a desire to use one, and the participants were concerned that this could result in digital exclusion.

Beyond owning a smartphone, a salient point was made about the type of smartphone and the impact that this could have on accessibility for patients and that the app should be available to Android as well as Apple users. Also, providing support to use the app may be required, following the experiences of a new booking system that had been launched.

*It was just, sort of, rolled out. And a lot of my clients didn’t… You know, were quite scared of how to use it. Like you said, there wasn’t that support, so they couldn’t log on and stuff. And a lot of the time, I used to spend the first one or two appointments that I had with them not doing any clinical work at all - but actually showing them how to set up the attend anywhere. Showing them how to log on* (R3).

Participants in all focus groups also discussed that for some patients, the app would be a challenge to use, from both a cognitive as well as physical viewpoint. For some patients, using a smartphone, or tablet for example, could be prohibitive due to the nature of more advanced PD, even if barriers around fine motor skills (to input the data) were able to be overcome with support such as voice recognition.

*Even using a tablet or a smartphone might not be the easiest thing in the world. You know, it’s okay if you’ve maybe got voice, you know, activation - but, again, we know that voice can be difficult with Parkinson’s* (R1).

Participants highlighted how the data was collected from the app could be a barrier to implementation, in terms of gaining permission from NHS Trusts to comply with information governance, but also for the app to be able to communicate with multiple IT systems, such as those within the NHS. Additionally, not all clinicians would have IT access for all appointments, therefore may not be able to use the data from the app in all situations. While printing off the information prior to an appointment was a workaround, for example when working within the community, it was not without drawbacks.

*The printout would be great, but that would then involve them having to print it out at home, and bringing it in physically, if they can’t get it to you beforehand. So, the practicalities, if you were community based I think would… May be more challenging than if you were in a clinic setting* (R4).

Throughout the focus groups, there was a clear consensus that recording falls was important, that the accuracy of the recording was key, and that the iFall app could play a positive role in enhancing the understanding of falls in clinical practice. Although the participants highlighted implementation challenges for the iFall app, there were no concerns raised about falls recording or using the iFall app to record falls *per se*, due to the importance of clinicians understanding falls. However, one participant did highlight the possibility that falls recording, not specifically the iFall app itself, could introduce an unintended consequence for some patients. For some patients, falls could be linked to a fear of falling, therefore, asking the patient to increase their focus on falls, could have the unintended consequence of increasing their fear of falling.

*Her main reason for falling is the psychological fear of falling. She’s so anxious, so focused on it. Because every time we get together, we’re talking about why are you falling? What’s going on? How are you doing? But sometimes that self-reporting can get her so focused on the fact that she potentially has a fall. And I suppose it’s getting that balance between if you’re seeing the pattern going, how would you then use it? And… And is that self-reporting actually making her more aware, but not in an insightful, let’s do something about it, way* (R3).

### The Future of iFall in Clinical Research

Participants considered how the app could be utilised within clinical research in the future. Research response rates were discussed by participants, citing different experiences using paper diaries, postcards and prompts. Participants felt that paper diaries were limited because the participants would forget to fill them out, forget to bring them, or fill them out retrospectively. A point was raised by one participant, who reported a 90% response rate in a study involving PwP using postcards and prompts but felt that the app could improve upon this response rate. As within clinical practice, the respondents felt that the app could increase the accuracy of falls reporting, and use the data to investigate patterns between falls and changes in medication for example:

*It would absolutely solve problems that we have with subjective reporting... …Because of the… Because of the variability in response to medication. And… How that might attract with their falls* (R2).

Adaptability within the app, was considered important so that it could be tailored for a research or clinical role, where making a distinction between the two was important. As with the discussion relating to the clinical use of the app, there was also consideration about ensuring that the app was inclusive, did not discriminate against participants that did not wish to use an app, meaning there should be alternative options for those participants. In terms of suggestions for the app, there was limited discussion. There was a request for the data to be provided within code, and a suggestion to increase the detail of information collected about medication, as opposed to just baseline measures- with the aim of being able to see the impact of medication changes or interactions with other medications on falls. It was also suggested that the iFall app needed to be assessed against current practices, to indicate that it was superior, suggesting that further research would therefore be required:

*From my perspective, I suppose, it’s definitely going to be hopefully more accurate than the falls diaries that we’ve got, but you’ve still got those as the gold standard. So, you’re going to have to show that your method is going to be more reliable than that, potentially. So, you might need to do a… Even a study looking at somebody using diaries and somebody using the application* (R1).

The topic with the biggest consensus within the study was the importance of having PwP involved with research. All focus groups were in clear agreement about this notion. Involving PwP from the design through to the implementation of the research was key, given people with Parkinson’s lived experience and important contribution:

*I think it’s brilliant. And, you know, we… It’s obviously… It’s become much more common with research studies, recently…*

*…So, it’s massively important, because you can just give the real perspective* (R5).

### Future Developments

The participants discussion within this theme focused on the future of the app, the potential benefits, and some of the considerations that they felt were important to be contemplated when further developing the app. There were two subthemes within this theme: 1) Future additions to the app and 2) further development of current app functions.

#### Future Additions to the App

To improve functionality, it was suggested for the addition of allowing the app to upload photographs, so that users could provide evidence where they fell, or the environment they were in, to help support the clinicians. Also supporting functionality, but also accessibility for patients, was the idea of adding in voice recognition to the app. Voice recognition could make inputting data easier for PwP and also giving carers the opportunity to easily input information.

*I think for me, I think it’s definitely the voice recognition part of it. You know, and being able to do that. Because, you know, lots of people… If you’re… If you’re off, or if you’re really shaky or anxious or dyskinetic. Then you might struggle, you know, with that. And you’ll just get frustrated. So, it is, you know… You know, being able to, you know, record… But again, it’s about how that picks up somebody’s voice. As well. So… Yeah, it’s just… It’s just thinking about, you know, all the complications with Parkinson’s* (R1).

Providing opportunities to record the emotional impact of falls, as well as providing scope to help patients reflect on falls were considered as further possible additions to the app. Participants were aware of the multifactorial impact falls can have and suggested the app to have the ability to capture this information.

*You know, your falls - all the outcomes were all physical outcomes. As in, you know, bruised, injured, hurt myself…I just wondered whether there should be some non-physical ones around, sort of, fear, dignity, anxiety… Or frustration* (R6).

Using the app to help avoid future falls was considered important, and a method of doing this was having an app that could be capable of giving feedback to patients through the recognition of falls patterns:

*With the app, say if somebody had no falls for a period of time and they were using this app, and suddenly started falling - is there something that’s generated in the app to sort of say, you really need to go and contact your Parkinson’s specialist nurse? As, you know, a sudden onset of falling? Because I just…I really think what [Name] just said there, in terms of flagging the near misses…You could be at those sort of people where we could be intervening early, before falls happen. But a sudden onset of falls would concern me. Is there some built in intelligence?* (R6).

Ensuring that the app was able to record additional signs or symptoms related to falls was considered as a worthwhile addition, as this could help provide clinicians with insight about causes of falls, to help reduce them occurring in the future. The ability to record urinary symptoms, dizziness or constipation for example, could possibily aid clinicians in supporting patients, and an inability of the app to do this at present, was considered by one participant as a negative. The participants were keen to discuss how further additions to the app could support clinicians when using it, and this was in the form of a range of suggestions/hints. From a user-friendly perspective, adding in hints about what questions to ask patients was considered, not only to provide an aide-memoir, but also to help develop more junior clinicians when assessing patients, with helpful reminders or hints about what questions could be asked. There were also relativity simple additions suggested, such as providing a comment box, where additional information or clarification could be provided about a specific aspect of a fall, for example about how the patient felt prior to a fall, or adding in outcome measures for the clinicians to populate within the app.

Although the potential benefits of each suggestion were highlighted by the participants, they also discussed how that some suggestions could be difficult to implement. For example, the inclusion of a falls detector into the app, or an ability for live feedback from the app for clinicians to access at any time were highlighted. However, the participants felt that the detector could be liable to false reporting, or increased cost, whereas the live feedback would be inhibited by logistical challenges or hadn’t been considered by the developers.

#### Further Development of Current App Functions

Gaining detail about a fall was discussed as a key benefit of the app, but it was also an area where participants wanted to be able to record as much detail as possible, as seen within the previous subtheme. However, beyond having options to add details about symptoms for example, which wasn’t an option in the app, they wanted to be able to expand the detail about what was included, such as if alcohol had been involved in the fall, or other information:

*I would like to know whether they needed help to get up. That would be the other key thing from a…From my point of view. Could they get up themselves, or did they need help? That would be another thing that I would… I would have on that* (R4).

While information about patient medication was included in the app, there wasn’t an ability to add if there had been any changes to the medication since the app was first used by the patient, which was something picked up on by the participants to change. Whilst having the app available on mobile was supported, the participants also suggested ensuring that it was available on a tablet to help support useability of the app.

Finally, a topic that generated in depth discussion was in response to the possibility of including an alert function, which would trigger a response if a fall was detected, such as a call to the user, to check on their wellbeing. The participants highlighted a range of factors that they felt needed to be considered, prior to any implementation. The ethics, resources, logistics, protocols, and unintended consequences were all discussed. Making a distinction between the app being used for research and clinical practice was highlighted, particularly relating to research ethics and protocols. In the context of research, if a fall was recorded, the participant was required to contact the researchers to report it, but if they were admitted to hospital, the research team were notified by the hospital:

*Yeah, yeah. I think that call-back feature would be really important, to make that very clear that it’s a call back to give you more information, not as a call back as a health provider. That would be important for that* (R4).

Finally, having clarity about the process was considered important due to the possibility of unintended consequences of having a system that triggered an alert with every fall. It was highlighted that if the app was to be used this way within clinical practice, it would require further consideration, due to the possibility of overburdening health systems, as well requiring a clearly defined role to ensure that the services were working within their scope of practice.

*Yeah, the only thing about that is I would say we’ve got to be really careful, because, you know, in a service of 2000 patients, if I had a... You know, a trigger for every time somebody fell, that is all I would be doing all day, every day* (R1).

Please see Supplemental File 2, for additional supporting quotes for each theme.

## General Discussion

The aim of this study was to investigate the views of healthcare professionals with clinical and research expertise, about a novel smartphone application, designed for falls reporting in PD. There was a clear consensus amongst participants that the iFall app could play a valuable role within clinical practice and research.

This study highlighted key issues with current falls reporting, including a lack of accuracy as well as compliance in the frequency of paper diary completion, which has been reported previously.^10,12,18^ Gaining detailed information about a fall during an appointment, was also highlighted as a challenge for clinicians, due to the nature of PD, where memory and communication is inhibited.^9,19^

The participants highlighted what they viewed as important for falls reporting, and how the app could address the current limitations of falls reporting. Understanding healthcare professionals’ requirements for falls reporting provided insight into the potential benefits of the app. For example, accurate recording of the number of falls, and the environment within which the fall occurred, was viewed as important to help patient management and reducing appointment time, as gaining details about a fall using current practice is time consuming. The participants considered that falls information could support multidisciplinary interaction and communication (for example with occupational therapy), which is a key aspect of providing comprehensive care within PD.^20^ Challenges for PwP and their carers relating to communication with health care professionals, as well as short duration of appointments has been cited,^21^ which highlights where the app may play a role by improving communication and saving time within appointments. The use of digital technology such as smartphone apps for healthcare purposes is growing in the UK NHS system and beyond. Other apps on the market aid with other health conditions, such as diabetes,^22^ and the iFall app may provide a supporting tool for falls in Parkinson’s to aid clinical practice.

The visual appearance of the app was favourably viewed by participants, and feedback suggested minimal development was required. This is likely due to the fact that the app was coproduced by PwP,^12^ which was described as highly important by the participants. It is encouraging that this has been recognised by researchers and clinicians, and an important message to send,^23^ particularly where involving PwP in research has improved how technology has been adapted previously in research.^24^

Importantly, participants highlighted potential barriers to implementation of the app. Ideally, participants wanted to have data from the app available prior to an appointment. However, barriers relating to information governance and synching the app to systems, such as within healthcare IT infrastructure, were seen as problematic. This, alongside issues regarding the interpretation of a fall/near miss, were viewed as most pertinent. Challenges of implementation or integrating digital tools or multiple systems within NHS has previously been discussed^25^ as well as the issues relating to the interpretation of a near miss.^12^ While these barriers are not necessarily specific to the app itself, but wider systematic or definitional discussions, they are important to be taken into consideration for future development and piloting of the app.

Specific to research, the app was viewed positively and appeared to have less implementation barriers compared to clinical practice. Limiting subjectivity with accurate data collection was a key benefit, but in order to verify the accuracy and reliability of the app, investigating the difference between current falls reporting practices and the app was suggested. Adaptability was also important for the participants, as was gaining app code, so they could tailor the app for different research questions/needs.

Digital inclusion was viewed as important, to ensure the app is accessible to all, unhindered by the operating system, or by disease severity. Piloting will be important to understand if the drawbacks of current falls reporting, such as patients forgetting to record falls, are overcome, improved or remain the same with the app. Digital health literacy is an issue impacting on all patients, not just PwP^26^ and therefore, should not become a barrier for PD patients using a smartphone app, and increase existing health disparities.^26^ Therefore, future research should consider the impact of digital health literacy when implementing future smartphone technology. Participants highlighted experiences of technology being rolled out too prematurely, which negatively impacted clinical practice. Therefore, in conjunction with piloting, implementation should be guided using the Digital Technology Assessment Criteria (DTAC).^27^

A range of suggestions were made to improve the app in its presented form, which included additions or refinement. Providing options to record further details about a fall was requested, such as patient’s reflections, injuries, symptoms or emotions upon a fall, to aid avoiding future falls, as were options to record medication changes. Prior work has investigated how PwP reflect on falls to understand why they fell,^28^ the significant emotional impact of falls for both PwP and carers, which can lead to concealment of falls,^21^ while underreporting of injuries in falls (in older adults) has been cited.^29^ Caregivers have also recognised a link between medication and falls.^21^ Given these findings, it highlights how the app may be used to support PwP, by using the data to guide conversation around falls prevention strategies as well as providing initial insight into how the patient is coping emotionally. All of these options would be simple to change within the app and indicates the apps adaptability.

Although considered more ambitious, there were suggestions to investigate if the app could have a built-in falls detector, or the ability for clinicians to have live feedback about patients. It is likely that developing accurate falls detectors is challenging, due to a lack of consensus on algorithm analysis,^30^ or due to the aforementioned information governance issues, making these difficult to implement. Developing artificial intelligence to help look for falls patterns, which would trigger the app to advise patients to seek clinicians’ input was also highlighted. However, this would require significant technological development. To support digital inclusion, adding voice recognition or allowing carer input options were suggested, allowing those with more advanced PD to use the app, or at least allow input from a carer. While speech recognition using artificial intelligence has been investigated within PD, it may not be ready for clinical practice,^31^ however in contrast, allowing carer input would not be a challenge for the app.

Interestingly, while many of the suggestions to improve the app involved adding new options, this contrasted with the notion that there was a danger of adding too many functions to the app by the participants. However, it is not clear where the balance between enough, or too many functions of the app lie. This would require careful consideration when piloting the app further, particularly by including the views and experiences of PwP. It was clear from the study, that careful piloting of the app is required, to minimize the impact of digital exclusion, health literacy and avoid unintended consequences (such as overburdening services with falls detection), while recognising that some of the barriers are specific to a UK context and its extant healthcare system.

### Strengths and Limitations

This work was co-designed, co-developed, and co-conducted involving PwP, building upon the prior PwP coproduced app.^12^ This study included highly experienced and active clinicians and researchers, from different professional backgrounds, therefore limiting status differentials^32^ and providing divergent perspectives.^32^ However, not all healthcare professions were represented within the cohort (such as occupational therapists), therefore future work should encompass a wider range of professional perspectives. Despite this, there was a consensus regarding the potential benefits of the app across professional backgrounds. Typically, focus groups consist of six to eight participants,^33^ whereas this study included two to five per focus group, which is justified when participants are highly engaged^33^ (such as professionals discussing practice). Methodologically, this study utilised independent researchers using multiple coding, providing independent analysis verification^34^ and code refinement.^35^ During the analysis, frequent debriefing sessions, peer scrutiny and the use of reflective diaries were utilised to improve trustworthiness.^36^ The use of framework analysis provides a clear audit trail of the analysis.^37^

## Conclusion

This is the first study to gain healthcare professional feedback on a smartphone app for falls reporting. Healthcare professionals considered the app, in its current form, to be clear, simple and easy to use, while having the potential to play a viable role within clinical practice and research. Participants identified potential areas for further development, which will require input from PwP. Implementation of the app will require consideration of current barriers that may inhibit successful use in clinical practice, particularly if clinicians desire the information from the app to be available prior to clinic appointments.

## Supplemental Material

Supplemental Material - The Views of Healthcare Professionals on iFall, a Smartphone Application for Falls Reporting in Parkinson’s Disease: A Qualitative Study Supplemental Material for The Views of Healthcare Professionals on iFall, a Smartphone Application for Falls Reporting in Parkinson’s Disease: A Qualitative Study by Michael C. Kelly, Jenni Naisby, Jill Wales, Elaine Webster, Gerry Standerline, Gill Barry, Annee Amjad, Jason Moore, Natasha Ratcliffe, Alan Godfrey, and Rosie Morris in Journal of Geriatric Psychiatry and Neurology

Supplemental Material - The Views of Healthcare Professionals on iFall, a Smartphone Application for Falls Reporting in Parkinson’s Disease: A Qualitative Study Supplemental Material for The Views of Healthcare Professionals on iFall, a Smartphone Application for Falls Reporting in Parkinson’s Disease: A Qualitative Study by Michael C. Kelly, Jenni Naisby, Jill Wales, Elaine Webster, Gerry Standerline, Gill Barry, Annee Amjad, Jason Moore, Natasha Ratcliffe, Alan Godfrey, and Rosie Morris in Journal of Geriatric Psychiatry and Neurology
