# Supplementary material for: The Views of Healthcare Professionals on iFall, a Smartphone Application for Falls Reporting in Parkinson’s Disease: A Qualitative Study
Source: J Geriatr Psychiatry Neurol. 2025 Feb 1;38(5):405–16. doi: 10.1177/08919887251317728 (PMC12202823; doi:10.1177/08919887251317728)
Supplement: Supplemental Material - The Views of Healthcare Professionals on iFall, a Smartphone Application for Falls Reporting in Parkinson’s Disease: A Qualitative Study [file sj-pdf-1-jgp-10.1177_08919887251317728.pdf]

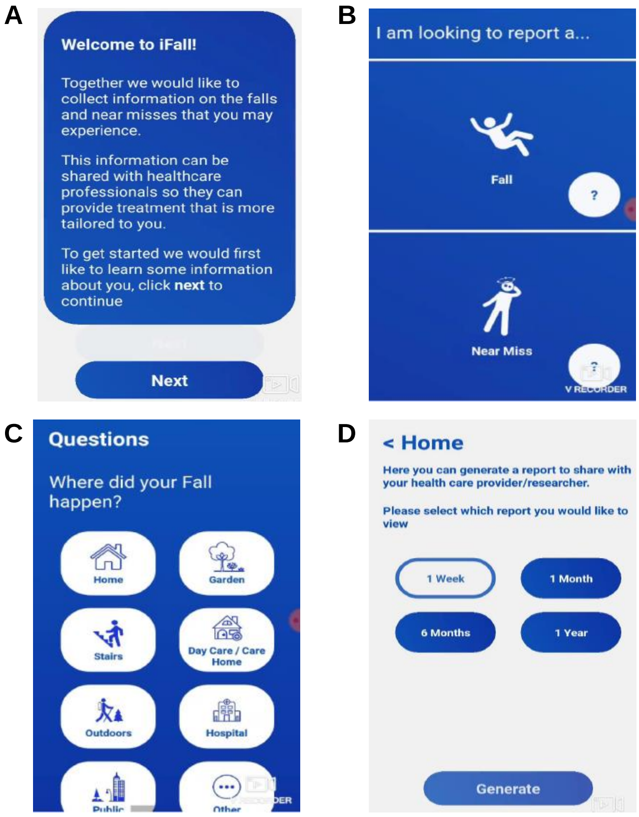

Supplementary Figure 1. Screenshots of the iFall prototype. **A)** welcome screen, **B)** Report Screen, **C)** Fall Details and **D)** create a falls report

1. Wales J, Moore J, Naisby J, et al. Coproduction and Usability of a Smartphone App for Falls Reporting in Parkinson Disease. *Physical Therapy*. 2023;104(2)doi:10.1093/ptj/pzad076
