# Supplementary material for: The Views of Healthcare Professionals on iFall, a Smartphone Application for Falls Reporting in Parkinson’s Disease: A Qualitative Study
Source: J Geriatr Psychiatry Neurol. 2025 Feb 1;38(5):405–16. doi: 10.1177/08919887251317728 (PMC12202823; doi:10.1177/08919887251317728)
Supplement: Supplemental Material - The Views of Healthcare Professionals on iFall, a Smartphone Application for Falls Reporting in Parkinson’s Disease: A Qualitative Study [file sj-pdf-2-jgp-10.1177_08919887251317728.pdf]

Theme	Subtheme	Illustrative quotes
1) Applicability to clinical practice	Ifall could enhance clinical understanding of falls in PwP	*So, the sort of people that are giving quite a hazy story, or a quite hazy recollection…whether it be due to cognitive issues or whatever else - struggled with the concept of the diary. The ones that were generally better historians anyway, would keep the diary or keep the calendar and make notes. But they probably weren't the ones that you needed to target. So, it's the ones that struggled with the recall, and then they would struggle to remember to fill in the diary. Or it would be, like you said, a week later. So, it wasn't as useful as… as you would hope it would be (R4).*<br><br>*But I suppose then that leads back in terms of the data sharing of this. I need this in advance of them coming in, so I can make them… use of it. Rather than bringing them in and then I feel socially awkward while I'm looking through it. While they're sitting there doing nothing in front of me. So, that that would be my thoughts, [Name]. But I definitely like the app. I think it's fantastic (R6).*<br><br>*Yeah, because you'll ask somebody... You know, how's your walking been - has it changed? Has it altered at all? Have you had a fall? And, yeah, it's very rare that you'll get somebody that, you know, can give you any details (R1).*<br><br>*And where where you fall in and they give you a general kind of answer and then you go out to their home and they've got five rugs leading along this path and and hardly any handrails or just and just things everywhere. And you just think it's not actually entirely your Parkinson's. There's caused these falls. It's the environment you're in as well. So asking where where it is. And like you said, the feedback you had specifically where in the home (R7).*<br><br>*Obviously in research you need it as an outcome measure - number of falls - and in clinical practice you would also obviously look at how many falls they have in a day. But I think, for me, it's kind of the patterns of how they're falling, where they're falling, that are just so vital. Because that… When, kind of, you first told me about this app, the idea that someone could come in and… You know, you'd show me one of the reports… With that kind of circle of where are the majority of the falls happening - are they happening at home, are they falling forwards or backwards… …But I think, you know, just getting a bit more detail. Like you said, you know, if it's in the home, I would want to know was it in the bathroom? What were they doing? Were they getting…? But maybe that's just that this starts that discussion, rather than getting the app to everything. I suppose, that's the thing, isn't it? We don't want it to replace that discussion, but we want it to stimulate that (R3).*
	Implementation challenges of iFall	*That's a good question. I haven't read much about near misses. So, I think it's definitely under… Under reported, or under studied. These things, so… Having this would give us more insights into it, for sure. But then, of course, like [Name] said, it's a little bit tricky - what your definition is exactly. Not everybody interprets that the same way, so…(R2).*

		And I think the Android-Apple thing is quite interesting, because we sort of assumed that everybody would be using Apple when we started using a lot of… Of digital tech in our rehab. When, actually, because… Especially in kind of… In [location], where there are some very unaffluent areas, as such… You know, actually going for, you know, a lot of people will have the tablets or the phones that are actually the cheaper versions, which aren't Apple and such like. And it's actually got to the point now where… When we're doing rehab in department, I've actually sourced a load of random tablets that I've got free off places, which have got different platforms on them (R3).
2) The future of iFall in research		*So, I think hopefully it'll give us much more accurate data - both clinically, but also for research purposes. And I think hopefully we'll identify, you know, the reasons for falling more accurately from this kind of, you know, information than we would have done from just pure recall alone (R1).*<br><br>*And then the other thing was about the circumstances in which they fall. I think we have research tools that do understand this, but those are also subjective questions about where they feel like they're going to fall, etc. Or we tend to understand, based on those reports, what aspect of their postural control or balance might be effected and those kinds of things. But if we had more direct reports from here, that would help us to really narrow down, and to say, okay, these falls were related to this, and these falls were related to that. So… In terms of the triggers for falling, if we can kind of… Cluster them into, sort of… Is this something from outside? Is this something from inside? Is this a particular kind of movement that we're looking at? (R2).*<br><br>*it's vital. You know, it really is. It's the most important thing that there is, you know …You know, what we want to do is try and improve the care that we provide for people with Parkinson's and…You know, our care partners. And the only way we can do that is with you, listening to you. You know, and working, you know, very closely with you - as you're the experts, not us (R1).*
3) Future developments	Future additions to the app	*Maybe there's a point to just a bit of self-reflection rather than the emotion, but more like what would you? What would you do differently? Or is there something you do differently? Well, it's hard, isn't it? Cause obviously, if they're dizzy or it's not, that's not their fault and they need to, but it might be like I need to bring the doctor. So yeah, that'd be interesting (R10).*
	Further development of current app functions	*Yeah, I guess… So, from [Name], just around… You know, the use and practice, I think that would have to be very clear as to what purpose they are serving. Whether it was just… Because, obviously, if they're filling in a falls diary, they should have a list of things to do in the case of a fall, but it wouldn't be a direct contact to the researcher or the team that's involved. That would be the issue for me, that I think would have to be very, very clear if you're going to start using any study. And, like you say, to get through ethics, you're going to have that very much written in (R4).*<br><br>*So, some sort of a traffic light system (R6).*<br><br>*Perhaps another aspect... Of course, I'm outside of the NHS - but another aspect that might be useful is that if it*

		actually triggers some sort of red flag, you know. If there is suddenly an increasing number of falls, or a first fall, for example - that it somehow gets signalled to the healthcare providers. Because that's one of the flags for a differential diagnosis. For example, progressive of Parkinson's and those kinds of things. So, I would rather see it before they came into the clinic, and maybe even on its own - that it triggers some sort of, yeah, response (R2).<br><br>*Plus multiple other people managing that. And it's about, you know, understanding what needs to be done immediately. We've got to remember that within specialist services, we're not an emergency service. So, I'm not there to suddenly, you know, check to see somebody if they've had a fall. Whether they've then fractured or done some damage to themselves, they're going to have to go to emergency service. And what we don't want to do is have people thinking, okay then, fine - it's triggered to the... You know, the Parkinson's team service. They'll pick it up. They'll manage that. Because that's not going to happen. You know, we're not there 24/7. You know, Monday to Friday, and we've got really limited resources. So, the problem is how are you then going to.... You know, what we don't want to do is raise that expectation. What you might do, though, is have within your app, built in to say - okay then, if you've had this many falls, or if you've had these symptoms... Blah-blah-blah... Loss of consciousness. You know, whatever else it is. Then you need to contact this. And you might be a priority thing. So, if you've had a loss of consciousness, contact your GP or go to A&E. If you've got this, then go to there. But what we don't want to do is automatically refer people back into the Parkinson's service, because just not sustainable. Because that's just one of many symptoms people could present with. And if everybody contacted us with, you know, all their symptoms... You know, it's just... It's just unsustainable. You know, we just couldn't manage that. We would just be swamped and cry in a corner (R1).*<br><br>*Yeah, no - I agree. I was not referring to, like, emergency care. But more about, sort of, flagging for the specialist so that they can address it the next time or something. Maybe you can each person if they want that. Obviously because people who don't want it would be pretty annoyed if somebody shows up at their house. So… Like, if that's a feature, then maybe that could be an optional feature for people who would like to get support if they fell. And then they could be automatically triggering some sort of emergency service (R2).*<br><br>*Yeah, I mean, I'm wondering - in the community, often people will have what we call Care Call. So, you know, they might have a button or a pendant or whatever else. Whether there is another option - because often they can be linked in with mobile phones, for example, to support somebody from getting off the floor. Getting checked out. You know, so whether you have something. But, again, it's just making it really clear, you know… You know, who's going to be the most appropriate person to call. Because obviously if somebody has, you know, fallen and hurt themselves, you really need them to get to be seen and assessed medically. And get them into hospital to be looked at, you know (R1).*
